# Predictors of Response to Growth Hormone Therapy in Children with Idiopathic Growth Hormone Deficiency: A Narrative Review

**DOI:** 10.3390/children13040545

**Published:** 2026-04-14

**Authors:** Ignazio Cammisa, Giulia De Fortuna, Eleonora Rulli, Donato Rigante, Clelia Cipolla

**Affiliations:** 1 Department of Pediatrics, San Giovanni Evangelista Hospital, 00019 Tivoli, Italy; 2 Department of Life Sciences and Public Health, Fondazione Policlinico Universitario Agostino Gemelli IRCCS, 00168 Rome, Italy; giulia.defortuna01@icatt.it (G.D.F.); eleonora.rulli01@icatt.it (E.R.); donato.rigante@unicatt.it (D.R.); clelia.cipolla@policlinicogemelli.it (C.C.); 3 Department of Pediatrics, Università Cattolica Sacro Cuore, 00168 Rome, Italy

**Keywords:** growth, growth hormone therapy, growth hormone deficiency, childhood, personalized medicine, bioinnovative therapies

## Abstract

**Background**: Growth hormone deficiency (GHD) in childhood impairs linear growth and may affect body composition, metabolism, and quality of life; recombinant human growth hormone (rhGH) therapy improves outcomes, but response is highly variable, especially in idiopathic GHD (IGHD). **Objective**: To summarize current evidence on predictors of growth response to rhGH therapy in children with IGHD, focusing on clinical, biochemical, and treatment-related determinants. **Methods**: This is a narrative review dealing with studies assessing clinical, auxological, biochemical and treatment-associated factors that may influence response to rhGH in IGHD. **Results**: Early treatment initiation, baseline short stature, prepubertal status, and higher early height growth velocity are strong clinical predictors; biochemical markers, including GH peak, IGF-1, and IGFBP-3, provide complementary information. Modifiable factors such as GH dose, adherence to therapy, and therapy duration also influence outcomes. Integrated predictive models improve accuracy but require further validation. **Conclusions**: Growth response to rhGH in IGHD is multifactorial and could be individualized: early identification of suboptimal responders and personalized treatment strategies that integrate clinical, biochemical, and treatment-related data may optimize the final outcome. Future research studies should focus on validated predictive models incorporating genetic and molecular markers.

## 1. Introduction

**Figure 1:**
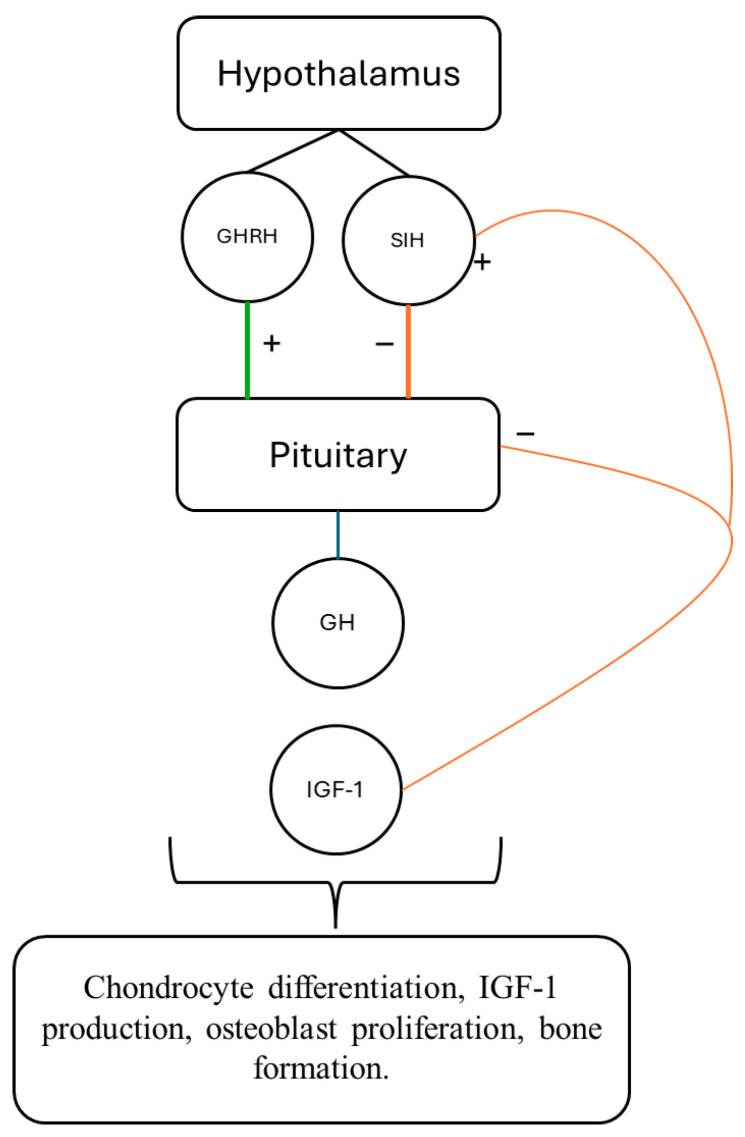
GH/IGF-1 axis regulation. Growth hormone (GH), Growth Hormone Releasing Hormone (GHRH), Somatotropin Inhbiting Hormone (SIH), Insulin-like Growth Factor-1 (IGF-1). Specifically, “+” should be used to indicate positive feedback (i.e., stimulation), while “−” should denote negative feedback (i.e., inhibition).

Growth hormone deficiency (GHD) in childhood is a well-established cause of impaired linear growth, with potential long-term consequences for final height, body composition, and metabolic health [1]. GHD is defined by inadequate secretion of growth hormone (GH) from the anterior pituitary gland, resulting in insufficient stimulation of hepatic and peripheral insulin-like growth factor 1 (IGF-1) production, which is essential for normal growth and ensuring overall anabolic processes, as illustrated in Figure 1 [1,2].

**Figure 2:**
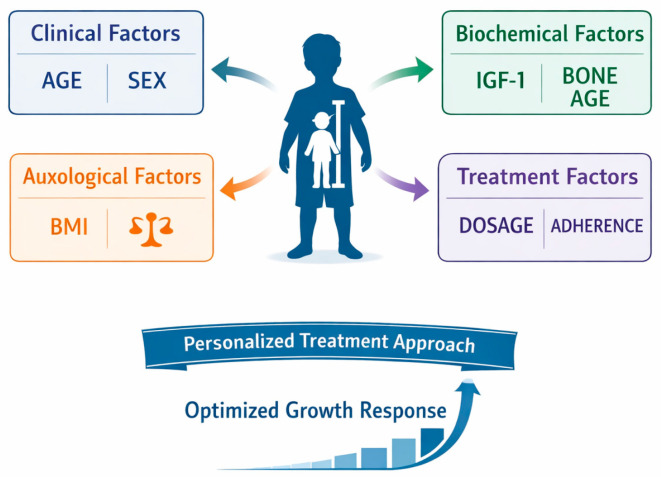
Factors influencing response to rhGH therapy in children with IGHD.

Clinical manifestations extend beyond reduced height velocity and include delayed skeletal maturation, increased adiposity, decreased muscle mass, and adverse lipid profiles, which may persist into adulthood if left untreated [1,2,3,4]. The diagnosis of GHD is based on a combination of auxological evaluation, biochemical assessment of GH secretion through stimulation tests, and measurement of circulating IGF-1 and insulin-like growth factor-binding protein 3 (IGFBP-3). Neuroimaging of the hypothalamic–pituitary region further supports diagnostic classification, allowing differentiation between isolated GHD and multiple pituitary hormone deficiencies [1,5,6,7]. GH stimulation tests remain the gold standard to confirm GHD. These dynamic tests evaluate the pituitary gland’s capacity to secrete GH in response to pharmacological stimuli. Despite their central diagnostic role, stimulation tests have important limitations, including inter-assay variability, limited reproducibility, and absence of universally accepted GH cut-off values, which continues to fuel debate regarding diagnostic standardization [5,6,7,8,9]. The introduction of recombinant human growth hormone (rhGH) therapy has profoundly changed the management of pediatric GHD, transforming a growth-limiting condition into a treatable endocrine disorder [10]. rhGH therapy has been shown to stimulate linear growth, improve body composition, increase bone mineral density, optimize lipid metabolism, and enhance quality of life [10,11,12]. However, the growth response to treatment is highly variable. This variability is influenced both by the underlying condition associated with growth failure—such as idiopathic GHD (IGHD), organic GHD (OGHD), Turner syndrome (TS), Noonan syndrome (NS), idiopathic short stature (ISS), or being born small for gestational age (SGA)—and by individual patient-related factors, including age at treatment initiation, severity of height deficit, body weight, sex, parental height, global duration of therapy, baseline hormonal values, and interactions with concomitant treatments [13,14,15]. While some patients achieve near-normal catch-up growth, others demonstrate suboptimal responses despite adequate dosing and good treatment adherence, highlighting the need to better identify determinants of GH responsiveness [16,17,18,19]. Understanding predictive factors of response to rhGH therapy is therefore essential for optimizing individualized treatment strategies. These factors may inform decisions regarding the optimal timing of treatment initiation, appropriate dosing regimens, and monitoring strategies and may allow early identification of poor responders, enabling timely therapeutic adjustments to maximize clinical outcomes [20]. Advancing knowledge in this area is crucial for the development of personalized medicine approaches in pediatric endocrinology and for improving long-term outcomes in children with GHD. Despite extensive literature on predictors of growth response to rhGH, clinicians lack a practical framework to integrate multiple patient-specific variables for individualized treatment planning, as represented in Figure 2. This review aims to fill this gap by proposing a stepwise evidence-based algorithm to stratify pediatric IGHD patients according to predicted response, supporting personalized therapeutic decision-making.

## 2. Materials and Methods

We carried out a broad review of the literature to explore factors that may influence how children with IGHD respond to rhGH. We searched PubMed using a range of terms related to rhGH treatment, growth outcomes, and relevant clinical variables such as age, sex, bone age, IGF-1 levels, and treatment adherence. We focused on studies involving children with IGHD who were treated with rhGH and followed regularly in an endocrinological setting. We excluded studies that were not in English, involved only adults, or did not provide meaningful endocrine data on growth response. Two reviewers independently screened titles and abstracts, then examined the full texts of studies. Any disagreements were discussed until a shared decision was reached. The studies that met these criteria were included in this narrative review, which aims to provide an overall picture of what is currently known about predictors of response to rhGH therapy in children with IGHD, summarized in Table 1. As this is a narrative review, we did not perform a formal study selection with a quantitative summary of study numbers, design, or time frame and we did not perform a formal quality assessment or meta-analysis. Moreover, study selection is non-systematic and based on expert synthesis. No temporal limits by year were applied, and studies of all designs were considered.

## 3. Clinical Predictors

Age at the start of therapy is one of the strongest determinants of growth outcomes. Multiple studies have demonstrated that younger children achieve higher catch-up growth, reflecting both a larger residual growth potential and a greater capacity for early skeletal adaptation. Ranke et al. compared two cohorts of children who initiated rhGH therapy at different ages: one before 3 years of age and the other between 7 and 8 years. The younger cohort showed significantly higher responsiveness to treatment, with a more significant height gain per rhGH dose compared with older children, highlighting the critical importance of early diagnosis and intervention in improving growth velocity and final height outcomes [21]. Ross et al. evaluated growth responses over two years in 3870 children and reported that prepubertal patients achieved the highest gains in height standard deviation score (SDS) (Year 1: 0.64 ± 0.53; Year 2: 1.15 ± 0.73), while children entering puberty exhibited slightly smaller increases (0.50 ± 0.41; 0.94 ± 0.53) and those already pubertal showed the lowest gains (0.48 ± 0.36; 0.95 ± 0.56) [16]. Similarly, De Ridder et al. observed a significantly higher increase in height SDS in children who initiated rhGH therapy before puberty compared with those who started treatment after pubertal onset [22]. This finding is consistent with the well-established concept that earlier treatment allows for a longer duration of growth before epiphyseal fusion and maximizes the biological response at the growth plate [23]. Quantitative analyses further emphasize the impact of treatment timing. Data from the American Norditropin Studies Web-enabled Research (ANSWER) Program registry confirmed that younger age at GH initiation was independently associated with greater gains in height SDS over five years of therapy, regardless of dose and sex [19]. Comparable results were reported by the LG Growth Study, which analyzed more than 1100 prepubertal children with IGHD and identified ‘age’ as one of the most powerful predictors of both first- and second-year growth response [24]. Collectively, these data underscore the clinical importance of early recognition of GHD and prompt initiation of GH therapy, particularly before the onset of puberty.

The role of sex as a predictor of growth response to rhGH therapy remains controversial. Several studies have reported that a linear dose–response relationship is more frequently observed in boys than in girls treated with rhGH, with males showing more significant increases in height SDS [20,25,26,27]. In contrast, other large cohort studies have found no significant sex-related differences in growth response among children with various etiologies of short stature [28,29,30,31,32,33]. Conversely, some reports suggest that female sex may represent a positive predictor of adult height gain following rhGH therapy, while boys may exhibit a reduced first-year growth response, possibly related to sex-specific differences in pubertal timing and progression [18,23]. Emerging evidence suggests that sex differences in growth response are not primarily mediated by circulating IGF-1 levels, but rather by differences in GH sensitivity, potentially driven by higher circulating concentrations of GH-binding protein and leptin in females [34,35,36].

Baseline height SDS represents a critical determinant of responsiveness to rhGH therapy. Children presenting with more severe short stature at treatment initiation generally achieve higher relative gains in height SDS following therapy, a phenomenon likely reflecting both regression to the mean and an enhanced physiological drive for catch-up growth. An inverse relationship between baseline height SDS and growth response has been consistently reported, with the most severely affected patients often demonstrating greater sensitivity to rhGH, particularly in the case of severe GHD [19]. Ross et al. showed that each 1-unit increase in baseline height SDS was associated with an approximate 0.2 reduction in Δ height SDS [17]. Similarly, Pozzobon et al. confirmed that lower height SDS at diagnosis predicted greater cumulative growth, especially among prepubertal children with severe GHD [18]. From a clinical standpoint, baseline height SDS is a simple and robust predictor that is essential for patients’ stratification and family counseling.

Height velocity (HV) during the early phase of treatment represents one of the strongest predictors of long-term growth outcomes. Lee et al. identified HV at four months as the most powerful predictor of Δ height SDS, exceeding baseline age, height SDS, BMI-SDS, and IGF-1 SDS [19]. Early growth acceleration likely reflects intrinsic GH sensitivity at the growth plate as well as residual growth potential. From a clinical perspective, assessment of HV within the first 3–6 months enables early identification of suboptimal responders and allows timely therapeutic adjustments. Bakker et al. demonstrated that children exhibiting a first-year height velocity of approximately −2 SD failed to achieve the expected growth response, highlighting that baseline age was a stronger determinant of HV-SDS than baseline height SDS [13]. The authors further suggested that a poor first-year growth response should prompt reconsideration of the initial diagnosis, raising the possibility of misclassified GHD or additional contributing factors, including unrecognized genetic disorders [18].

Consistent with these observations, multiple studies have shown that first-year growth response is a strong predictor of second-year growth and final adult height [37,38,39], with the initial year of therapy representing the period during which differences between good and poor responders are most pronounced [18]. Baseline body composition and genetic growth potential further modulate the response to therapy. Body mass index SDS (BMI-SDS) reflects nutritional and metabolic status, whereas midparental height SDS (MPH-SDS) serves as a proxy for genetic growth potential. MPH-SDS, calculated as [(father’s height SDS + mother’s height SDS)/1.61], was positively associated with GH response in the predictive models developed by Ranke et al., underscoring the strong influence of genetic target height on adult outcomes [21,40]. In the same cohort, weight SDS was also significantly correlated with treatment response. In a comparative study of children with idiopathic short stature, partial GHD, and complete GHD, MPH-SDS predicted more favorable growth outcomes in partial GHD and positively influenced height velocity in complete GHD, whereas baseline height SDS and BMI-SDS were stronger predictors in the complete GHD group. BMI-SDS also showed a consistent predictive role in partial GHD [41]. Consistent with these findings, Lee et al. reported a positive correlation between higher baseline BMI-SDS and Δ height SDS, supporting the concept that adequate nutritional status enhances GH efficacy [19]. Overall, these data indicate that the growth response to GH therapy is not determined solely by rhGH administration itself, but is modulated by intrinsic genetic growth potential—particularly midparental height and sex-specific parental contributions—as well as by age, sex, baseline auxological parameters, body composition, bone age, biochemical markers, and nutritional status.

## 4. Biochemical Predictors

Biochemical indices, particularly peak GH concentrations during stimulation tests and circulating IGF-1 levels, are associated with growth response to rhGH therapy; however, their role as reliable clinical predictors remains limited and context-dependent. Children with lower GH peaks, indicative of more severe GH deficiency, generally exhibit greater growth responses than those with partial deficiency, and several studies have reported correlations between stimulated GH peaks and growth outcomes [42,43]. Nonetheless, these associations do not consistently translate into strong predictive tools in clinical practice. For example, Albertsson-Wikland et al. showed that GH secretion profiles derived from spontaneous 24 h measurements may better reflect physiological GH dynamics compared to pharmacological stimulation tests, although their routine clinical applicability is limited [36]. Similarly, IGF-1 levels—both at baseline and during treatment—have been shown to correlate with growth velocity and are widely used for treatment monitoring. Early increases in IGF-1 SDS are associated with short-term height gains, and longitudinal changes may reflect GH sensitivity and adherence [44,45,46]. However, these relationships should be interpreted with caution, as correlation does not necessarily imply predictive accuracy at the individual level. In addition, the clinical utility of IGF-1 and related markers is significantly influenced by variability in assay methods, stimulation protocols, reference ranges, and timing of measurements, which may limit reproducibility across different settings. Cohen et al. reported that optimal growth is observed in patients with concomitantly higher IGF-1 and IGFBP-3 levels, suggesting a more complex interaction between these factors rather than a simple linear relationship with free IGF-1 [20]. Likewise, Lundberg et al. demonstrated associations between IGF-1, IGFBP-3, and height gain, particularly in children with isolated IGHD, with prepubertal IGF-1 levels influencing long-term outcomes [47,48]. The IGF-1/IGFBP-3 ratio has been proposed as an indirect marker of IGF-1 bioavailability; however, its clinical applicability as a predictive biomarker remains to be fully established [49,50]. Overall, while IGF-1, IGFBP-3, and GH-related parameters provide valuable supportive and monitoring information, their use as standalone predictors of treatment response is limited, and they should be interpreted within a broader clinical and auxological context rather than as definitive determinants of therapeutic outcomes.

## 5. Radiological Predictors

Bone age (BA), reflecting skeletal maturity compared to chronological age, is widely recognized as a critical component of auxological assessment in pediatric endocrinology. In children with GHD, BA is typically delayed relative to chronological age, and this delay has been associated with both severity of the deficiency and potential for catch-up growth during rhGH therapy. Although bone age is most commonly used to estimate residual growth potential, several studies suggest that it may also contribute meaningfully to treatment response [51,52,53]. Multivariate prediction models developed for children with GHD frequently incorporate BA alongside other baseline variables to improve the accuracy of first-year height velocity predictions. In a prospective Italian pediatric cohort conducted by Valle et al., baseline BA was included in a regression equation together with IGF-1 and early HV to estimate first-year growth outcomes, with lower BA values (indicating greater delay) contributing to higher predicted HV after rhGH initiation. In this model, bone age entered as a negative coefficient indicating that less skeletal maturity (greater delay) may be associated with a more vigorous early growth response, consistent with the concept that skeletal immaturity reflects greater growth potential [54]. Similar findings have been reported in studies of SGA children, in whom height SDS increased more significantly in those with bone age delay exceeding 2 years [55]. Large registry-based studies further support the inclusion of BA in predictive frameworks. Analysis of data from the LG Growth Study in prepubertal children with idiopathic GHD demonstrated that BA, combined with age, birth weight, baseline height SDS, BMI SDS, mid-parental height, GH dose, and first-year height gain, explained a substantial proportion of the variability in height SDS changes during the first and second years of treatment. This reinforces the idea that skeletal maturity, together with auxological and biochemical variables, enhances the predictive performance of growth models [56]. Beyond formal prediction models, the dynamics of BA progression during rhGH therapy also offer insights into treatment responsiveness. Children with IGHD often show a gradual advance of bone age over the course of treatment, paralleling improvements in height velocity; more pronounced BA advancement during early therapy may thus reflect effective GH action on skeletal maturation processes [53]. Although studies directly quantifying the independent predictive value of BA on long-term outcomes in IGHD remain limited, these observations collectively suggest that bone age delay and its evolution during therapy are integrally linked to growth response dynamics, warranting their consideration in individualized treatment planning and prognostic assessment.

## 6. Treatment-Related Predictors

Treatment-related factors, including GH dose, adherence to therapy, and therapy duration, are key modifiable predictors of the global growth response. Higher daily GH doses, within the recommended safety limits, are consistently associated with greater early height gains, particularly in children with severe GHD. Conversely, poor adherence significantly reduces treatment efficacy, with observational studies indicating that non-adherence accounts for a substantial portion of interindividual variability in growth response. Treatment duration has also been identified as a positive predictor of final height gain [18]. Accordingly, technological support, patient education, and systematic adherence monitoring should be integral components of GH therapy planning.

GH dose has emerged as one of the strongest determinants of early growth response. In a randomized clinical trial of 111 children with GHD, Cohen et al. demonstrated a clear dose–response relationship, with higher GH doses resulting in significantly greater height SDS gains over one year. Specifically, daily doses of 0.05 mg/kg and 0.1 mg/kg produced Δ HSDS of 2.1 ± 0.9 and 2.6 ± 0.9, respectively, compared with 1.1 ± 0.9 in children receiving 0.025 mg/kg/day [20]. Similarly, Ross et al., analyzing data from 12,683 children, reported that each 0.01 mg/kg/day increase in GH dose was associated with measurable increases in Δ HSDS (0.03 in females, 0.02 in males) [17]. Despite its strong effect on short-term growth, GH dose does not independently predict final adult height. Carel et al. observed that GH dose primarily accelerates early catch-up growth rather than determining ultimate stature [23]. This distinction underscores the importance of dose optimization to maximize early growth while avoiding overtreatment. GH dose also influences circulating growth factors, particularly IGF-1 SDS, with several studies confirming a positive association between administered GH dose and adult height in children with GHD [57]. Mechanistically, GH dose determines the magnitude of IGF-1–mediated anabolic activity, primarily promoting linear growth through stimulation of chondrocyte proliferation at the growth plate. Dose optimization must, however, carefully balance efficacy and safety, as higher doses may increase the risk of adverse metabolic effects. Higher GH doses have also been linked to greater pubertal height gain SDS compared with standard dosing regimens and represent one of the most important predictors of first- and second-year growth response [21,58,59,60]. Historically, injection frequency was considered a determinant of growth response [13,38]; however, daily administration (six to seven injections per week) is now the standard of care. Finally, treatment duration and adherence are critical for long-term growth outcomes. Suboptimal adherence markedly diminishes GH effectiveness. Carel et al. reported that children completing therapy achieved a mean height gain approximately 0.3 SD greater than those who discontinued prematurely, with each additional year of therapy contributing an estimated 0.2 SD to final height [23]. Pozzobon et al. similarly demonstrated a positive association between treatment duration and adult height, particularly in children with severe GHD, and Lonero et al. confirmed a correlation between therapy duration and near-final height (r = 0.114, *p* < 0.001), highlighting the importance of a sustained therapy and long-term adherence to optimize final growth outcomes [18,61].

Recent studies of long-acting growth hormone (LAGH) formulations have expanded the therapeutic landscape beyond daily rhGH regimens [62,63,64,65,66,67,68]. LAGH formulations include either native rhGH with temporary modifications to prolong its activity or somatropin analogues with permanent structural changes that extend their half-life. They require only one weekly subcutaneous administration and have been designed to reduce the burden of daily injections and potentially improve treatment adherence in children with GHD, reducing the injection frequency from 365 to 52 injections per year [62,63,64,65,66]. In recent years, several studies and multinational randomized controlled phase III trials have validated the efficacy, in terms of annualized HV and change in HSDS, and the safety of LAGH compared to daily rhGH. Moreover, it has been documented that switching from daily GH to weekly LAGH formulations maintains comparable HV and HSDS in pediatric GHD [62,63,64,65,66,67,68]. For instance, the heiGHt trial showed a statistically superior annualized height velocity (AHV) of 11.2 cm/year in children treated with lonapegsomatropin compared to 10.3 cm/year with daily rhGH, while in the REAL 4 trial, somapacitan achieved an AHV of 11.2 cm/year, comparable to 11.7 cm/year with daily GH. Similarly, the somatrogon trial reported AHVs of 10.1 cm/year for once-weekly somatrogon versus 9.8 cm/year for daily GH [69,70,71]. Even if current evidence supports the feasibility of switching pediatric patients from daily GH to weekly LAGH without compromising growth or safety, future studies have to validate long-term surveillance, optimal dosing, timing of IGF-1 monitoring, cost-effectiveness, and real-world efficacy.

## 7. Genetic Predictors

Genetic factors are increasingly recognized as important contributors to the variability in growth response to rhGH therapy in children with GHD, supporting a pharmacogenetic approach to treatment. While the role of clinical, biochemical, and treatment-related factors in determining the response to rhGH therapy is well established—accounting for approximately 40–61% of the variability—the contribution of genetic factors to growth outcomes remains less clearly understood [72,73,74,75]. However, there is evidence that variation in growth-related genes, such as the deletion of exon 3 in the growth hormone receptor (GHR) gene or specific polymorphisms in genes of the growth hormone axis (as SOS1), may impact response to GH treatment [72]. The study of these polymorphisms has relevant clinical implications, as demonstrated by findings showing that the presence of a GHR isoform lacking exon 3 (d3-GHR) was associated with a significantly greater growth response, with a 1.7- to 2-fold increase in growth acceleration compared to the full-length receptor. Functional analyses further revealed that GH signaling through d3-GHR homo- or heterodimers is approximately 30% more efficient than through full-length receptor homodimers [75]. Wassenaar et al. in a systematic review and meta-analysis of 15 studies showed that the GHR exon 3 deletion (d3) polymorphism is associated with a modestly improved response to rhGH therapy in children with GHD. Carriers of the d3 allele had slightly higher baseline height and experienced greater increases in growth velocity (about +0.5 cm/year) and height gain during the first year of treatment compared to those with the full-length genotype, especially in the case of lower GH doses and in older children [76]. However, studies in GHD cohorts have reported conflicting results, with some confirming improved growth velocity in d3 carriers and others showing no significant association, highlighting the heterogeneity of findings and the need for further studies [74,77]. Other genes involved in the GH–IGF-1 axis have also been implicated as potential modulators of treatment response. Genetic variation in the IGFBP-3 promoter region may influence response to rhGH therapy, with the −202 A/C polymorphism associated with treatment outcomes, independently of other factors [73]. At the same time, SOCS2 polymorphisms are associated with higher adult height in patients with GHD [78]. SOCS2, GHR exon 3, and IGFBP3 polymorphisms, together with clinical variables, explain a substantial proportion of variability in growth outcomes [78]. The presence of unfavorable genotypes across these loci was linked to poorer adult height, suggesting that integrated genetic profiling could help identify patients at risk of suboptimal response to rhGH therapy. For example, Garner et al. conducted a phase 3 trial, which studied pretreatment blood transcriptomic profiling, and documented that gene expression patterns before treatment reliably predicted first-year growth outcomes, with strong performance across multiple growth indicators [79]. Overall, while genetic markers hold promise for personalizing rhGH therapy, particularly in identifying the best responders, their clinical utility remains limited by inconsistent evidence, and further large-scale, prospective studies are needed to validate their role in routine practice.

## 8. Growth Prediction Models

Several integrated predictive models have been developed to estimate individual responses to rhGH therapy in children with GHD and related growth disorders. Early efforts in this field focused on multivariate regression models that utilized auxological and endocrine data to predict growth outcomes after one and two years of GH treatment. Constructed and validated models combined baseline height data, parental heights, spontaneous GH secretion profiles, and early growth measurements, demonstrating improved predictive accuracy when endocrine variables such as 24 h GH profiles and IGF-1 levels were included alongside auxological parameters [37,80]. A recent analysis using the LG Growth Study registry developed stepwise multivariate regression models for prepubertal children with idiopathic GHD, integrating variables such as chronological age, birth weight, bone age, initial height SDS, body mass index SDS, mid-parental height, GH dose, and first-year height gain, which together explained up to 76.9% of the variability in height SDS change during the first year and 84.1% during the second year of therapy [56]. In parallel, nomogram-based predictive tools have been established in large pediatric cohorts with diverse growth disorders, including GHD. These models use multivariate logistic regression with variables such as diagnosis category, age, height SDS, bone age minus chronological age, rhGH dosage, weight SDS, mid-parental height distance, and IGF-1 SDS to estimate the probability of a poor short-term response to GH therapy, demonstrating good discrimination and clinical usability in both training and testing sets [81]. Validation of prediction models in independent cohorts, such as in the GeNeSIS post-marketing study, has shown that these tools can explain a substantial proportion of variance in first-year height velocity (53–72%) across prepubertal and pubertal children with isolated GHD, supporting their utility for early identification of poor and good responders [82].

Collectively, these integrated predictive approaches highlight the value of combining multiple patient-specific factors—clinical, auxological, biochemical, and treatment-related—in estimating growth response to rhGH therapy. Although many predictive models perform well in their original cohorts, external validation is still limited, and their accuracy may differ across populations and clinical settings. Models derived from the LG Growth Study registry, for example, have been internally cross-validated and tested in independent cohorts such as GeNeSIS, demonstrating only moderate generalizability. Variations in patient characteristics, treatment protocols, and measurement methods across cohorts may affect performance, introducing the risk of overfitting and cohort-specific bias [56,81,82]. Therefore, these models should be applied cautiously outside their development populations, and further multi-center external validation is needed to confirm their wider clinical applicability.

Moreover, it may be relevant to analyze whether the contribution of these predictors differs according to the severity of GHD. Evidence suggests that children with more severe GHD generally exhibit a greater first-year growth response to rhGH compared with those with partial deficiency [83,84,85]. In this context, baseline IGF-1 appears to play a more prominent role in severe GHD, whereas auxological and constitutional factors—such as parental height, BMI, and age at treatment initiation—may have a greater impact in partial GHD [83,84,85,86]. Overall, these findings support a model in which the determinants of treatment response are not uniform across the GHD spectrum, with biological severity predominating in severe cases and a more multifactorial pattern emerging in partial deficiency.

Importantly, all the predictors should not be interpreted in isolation, as substantial interactions exist across domains. Clinical factors such as age, pubertal status, and baseline auxology influence biochemical responses, particularly IGF-1 dynamics, which in turn mediate the biological effects of GH at the growth plate. Similarly, treatment-related variables, including GH dose and adherence, exert their effects largely through modulation of IGF-1 levels and tissue sensitivity. Moreover, the relative contribution of each predictor may vary according to disease severity, with biochemical determinants predominating in severe GHD and clinical or constitutional factors playing a greater role in partial deficiency. These observations support a systems-level model in which growth response emerges from the dynamic interaction between multiple interdependent factors rather than from the isolated effect of single predictors.

## 9. Stepwise Algorithm for Predicting Response to rhGH Therapy in Pediatric IGHD

**Figure 3:**
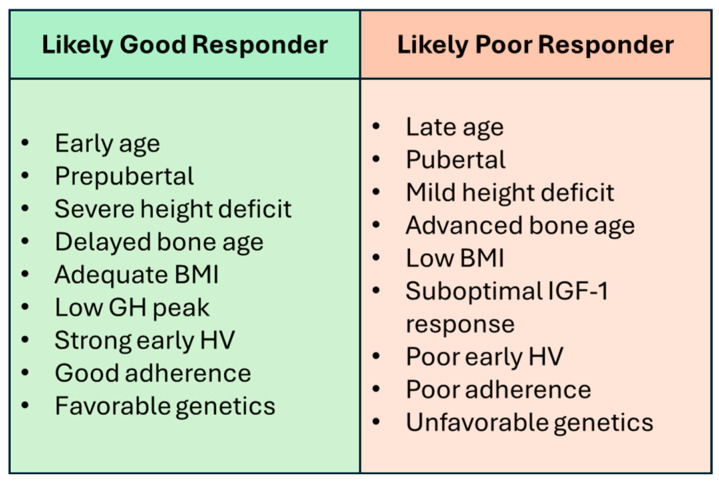
Stratification of response to rhGH in pediatric IGHD.

Effective management of pediatric IGHD depends on the ability to predict individual responses to rhGH therapy. Treatment outcomes are influenced by a complex interplay of clinical, biochemical, radiological, treatment-related, and genetic factors, which makes providing personalized guidance challenging. To address this, we propose a stratification framework that integrates these multidimensional predictors to classify patients as likely good or poor responders, as reported in Figure 3.

**Figure 4:**
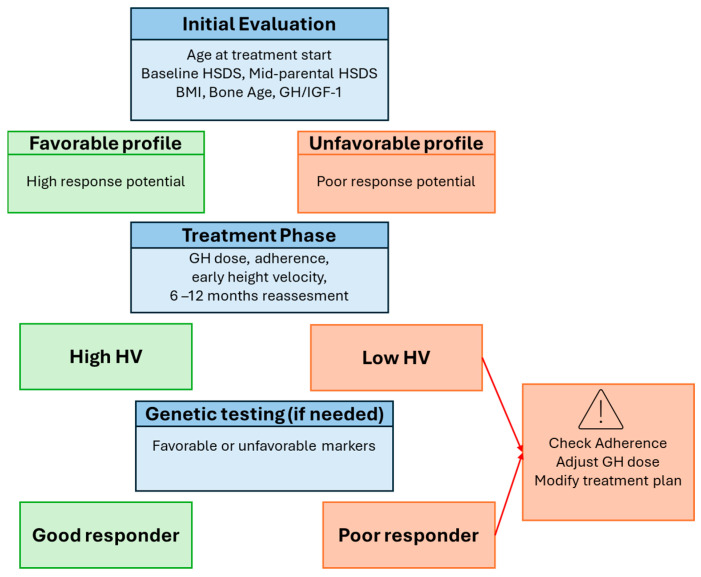
Stepwise algorithm for predicting response to rhGH therapy in pediatric IGHD.

This approach facilitates individualized monitoring, timely interventions, and optimization of therapeutic outcomes, supporting precision-guided rhGH therapy in children with IGHD. Furthermore, we propose a structured approach for clinical endocrinology practice, as reported in Figure 4, proceeding through sequential steps: initial evaluation, risk stratification into favorable or unfavorable profiles, close monitoring during therapy, and early on-treatment reassessment based on height velocity. By translating complex, multidimensional data into actionable insights, this framework enables precision-guided, personalized rhGH therapy in pediatric IGHD. From a practical standpoint, this framework can guide clinical decision-making at key time points:

- At treatment initiation, to identify patients requiring closer monitoring;
- At 6–12 months, to detect suboptimal responders based on height velocity;
- During follow-up, to support dose adjustment, adherence interventions, or further diagnostic evaluation.

Early identification of poor responders should trigger a structured reassessment, including verification of adherence, reconsideration of diagnosis, and, when appropriate, genetic testing.

## 10. Limitations

Despite extensive evidence on predictors of growth response to rhGH therapy, several limitations must be considered when interpreting the findings. First, as this is a narrative rather than a systematic review, the analysis is based on qualitative synthesis and critical interpretation rather than exhaustive study identification and structured comparison, which may introduce selection bias. Importantly, substantial heterogeneity across studies—including differences in diagnostic criteria, GH stimulation tests, assay methodologies, dosing regimens, and follow-up duration—reduces comparability and limits the generalizability of results. This variability is closely linked to a major clinical issue: diagnostic inconsistency in childhood GHD, where the lack of standardized GH cut-offs and the known limitations of stimulation tests increase the risk of misclassification, particularly in idiopathic and partial forms. Such misclassification may, in turn, affect the identification and strength of reported predictors. Furthermore, most available data derive from observational, retrospective, or registry-based studies, which are inherently subject to confounding factors (e.g., age, pubertal status, treatment adherence, and dosing strategies). These interrelated variables—along with the complex interactions between GH, IGF-1, and IGFBP-3—limit the ability to establish independent causal relationships and reduce the interpretability of findings. Additional limitations include the variability of identified predictors across different populations and the lack of external validation for many predictive models. Moreover, genetic and molecular determinants remain underexplored, and most studies primarily focus on height outcomes, with less consistent evaluation of other clinically relevant endpoints such as body composition, metabolic health, quality of life, and long-term safety. Overall, these factors highlight the need for standardized diagnostic approaches and more robust, prospective studies to improve the reliability and clinical applicability of predictive models.

## 11. Conclusions

Growth response to rhGH therapy in children with IGHD is a multifactorial and highly individualized process. Accumulating evidence indicates that treatment outcomes are shaped by the interaction of patient-related factors—such as age at treatment initiation, baseline severity of short stature, body composition, genetic growth potential, and biochemical markers—and treatment-related variables, including GH dose, adherence, and duration of therapy. Among clinical predictors, younger age at initiation, greater baseline height deficit, and a robust early growth response consistently emerge as the most reliable indicators of favorable outcomes. Biochemical parameters, particularly GH peak levels, IGF-1 and IGFBP-3 SDS, and their dynamic changes during treatment, provide complementary insights into disease severity, GH sensitivity, and treatment adequacy. Importantly, treatment-related factors remain modifiable determinants of response, highlighting the clinical relevance of dose optimization, adherence monitoring, and sustained treatment continuity. These findings support a personalized approach to GH therapy that integrates auxological, biochemical, genetic, and treatment-related data rather than relying on weight-based dosing alone. Future research should prioritize the development and external validation of integrated predictive models that incorporate genetic and molecular markers, standardized diagnostic approaches, and clinically meaningful outcomes beyond final height. Such efforts would advance precision medicine in pediatric endocrinology by enabling the early identification of suboptimal responders, facilitating timely therapeutic adjustments, and potentially improving long-term outcomes.

## Figures and Tables

**Table 1:** Summary of predictors of response to rhGH therapy in pediatric IGHD.

Predictor Category	Specific Predictor	Effect on Response	Strength of Evidence
Clinical	Age at treatment start (younger/prepubertal)	↑ Response	Strong
	Baseline height SDS (more severe deficit)	↑ Catch-up growth	Strong
	Mid-parental height SDS (higher genetic target)	↑ Response	Moderate
	BMI/Nutritional status (normal-high)	↑ Response	Moderate
	Sex (male vs. female)	Variable, context-dependent	Limited
Biochemical	GH peak (stimulation tests)	Lower peak → ↑ Response	Strong
	Baseline IGF-1/IGFBP-3	Low baseline + large early increase → ↑ Response	Moderate
Radiological	Bone age delay	Greater delay → ↑ Residual growth potential	Moderate
Treatment-related	GH dose	↑ Early height velocity	Strong
	Adherence	Poor adherence → ↓ Response	Strong
	Early on-treatment height velocity (3–6 months)	↑ HV → confirms good responder; ↓ HV → indicates poor responder	Strong
	Treatment duration	Longer duration → ↑ Final height	Moderate
Genetic	GHR d3 allele	↑ Growth acceleration	Moderate
	IGFBP3 promoter -202 A/C	Modulates response independently	Limited
	SOCS2 polymorphisms	↑ Adult height	Limited
	Transcriptomic profiling	Predicts first-year HV	Emerging

Strength of Evidence: Strong (multiple large cohort studies or meta-analyses), Moderate (smaller cohorts or some conflicting results), Limited (few studies, conflicting data), Emerging (promising new markers requiring validation). Body Mass Index (BMI), Growth Hormone (GH), Growth Hormone Receptor (GHR), Height Velocity (HV), Insulin-like Growth Factor 1 (IGF-1), Insulin-like Growth Factor Binding Protein 3 (IGFBP-3), Standard Deviation Score (SDS), Suppressor of Cytokine Signaling 2 (SOCS2), ↑ (increased), ↓ (decreased).

## Data Availability

No new data were created or analyzed in this study.
